# Thermal and Guest-Assisted Structural Transition in the NH_2_-MIL-53(Al) Metal Organic Framework: A Molecular Dynamics Simulation Investigation

**DOI:** 10.3390/nano8070531

**Published:** 2018-07-14

**Authors:** Roald Boulé, Claire Roland, Laurent Le Pollés, Nathalie Audebrand, Aziz Ghoufi

**Affiliations:** 1 IPR (Institut de Physique de Rennes)—UMR 6251, CNRS, University Rennes, F-35000 Rennes, France; roald.boule@gmail.com; 2 ISCR (Institut de Sciences Chimiques de Rennes)—UMR 6226, CNRS, University Rennes, F-35000 Rennes, France; claire.roiland@univ-rennes1.fr (C.R.); laurent.le-polles@ensc-rennes.fr (L.L.P.); nathalie.audebrand@univ-rennes1.fr (N.A.)

**Keywords:** NH_2_-MIL-53(Al), MOFs, molecular simulation, structural transition

## Abstract

Reversible structural transition between the Large (LP) and Narrow Pore (NP) forms (breathing phenomena) of the MIL-53(X, X = Al, Cr, Fe, Ga) Metal Organic Framework (MOF) is probably one of the most amazing physical properties of this class of soft-porous materials. Whereas great attention has been paid to the elucidation of the physical mechanism ruling this reversible transition, the effect of the functionalization on the flexibility has been less explored. Among functionalized MIL-53(Al) materials, the case of NH${}_{2}$-MIL-53(Al) is undoubtedly a very intriguing structural transition rarely observed, and the steadier phase corresponds to the narrow pore form. In this work, the flexibility of the NH${}_{2}$-MIL-53(Al) metal organic framework was investigated by means of molecular dynamics simulations. Guest (methanol) and thermal breathing of the NH${}_{2}$-MIL-53(Al) was thus explored. We show that it is possible to trigger a reversible transition between NP and LP forms upon adsorption, and we highlight the existence of stable intermediate forms and a very large pore phase. Furthermore, the NP form is found thermodynamically stable from 240 to 400 K, which is the result of strong intramolecular hydrogen bonds.

## 1. Introduction

The flexibility of soft-porous Metal-Organic Frameworks (MOFs) is probably one of the most fascinating structural features in the field of nanoporous materials in comparison with the most common materials such as active carbons and zeolites [1,2,3,4,5,6]. This flexibility is the result of the connection of the organic ligands with inorganic building units. For several MOF materials, this flexibility induces structural transitions triggered by several external stimuli such as light [7,8,9], electrical fields [10,11], mechanical pressure [12,13,14] and adsorption of gases [2,3,4,5,6,15,16,17,18,19,20,21,22,23]. Interestingly, it has been recently shown that this structural modulation (breathing, pore gating, ligand flip, etc.) allowed new adsorption and separation properties involving new areas in adsorption processes [2,3,4,5,6,15,16,17,18,19,20,21,22,23]. Among the flexible MOFs, the MIL-53 material is probably the most studied in this past decade with evidence of a guest-, thermal- and mechanically-assisted reversible structural transition between Narrow Pore (NP) and Large Pore (LP) forms [2,19,20,24,25]. Indeed, a reversible expansion/contraction of the pore dimension up to 40% in unit cell volume was observed whatever the external stimuli. At room temperature and atmospheric pressure, the steadier phase of the MIL-53(Al) material is the LP form, i.e., an orthorhombic structure with a large pore of 12 Å, inducing a unit cell volume of 1480 Å${}^{3}$. By applying an external (electrical field, mechanical pressure, etc) or internal (adsorption of gases) constraint, the MIL-53(Cr) is then thermodynamically and mechanically destabilized. To reach a new thermodynamical equilibrium, a structural transition occurs to trend toward a more steadier phase in line with this constraint. The pores of the MIL-53(Al) contract; the symmetry changes; and the volume decreases from 1480 Å${}^{3}$ to 1000 Å${}^{3}$: the NP form. When the constraint is released, the initial LP form is then recovered, emphasizing the reversible aspect of this structural transition: the breathing effect. Recently, the use of this family of materials was suggested for mechanical energy storage applications [12,13,14]. Whereas the MIL-53(Al) was intensively studied, the flexibility of the modified MIL-53 was less investigated. The structural behaviors of MIL-53 have been recently analyzed as a function of functionalized organic linkers [26]. It was thus highlighted that these chemical modifications had a deep impact on the breathing phenomena (reversible structural transition). Among functionalized aluminum hydroxo terephthalates (Al(OH)(BDC-X)) (with X -H, -CH${}_{3}$, -Cl, -Br, -NH${}_{2}$, -NO${}_{2}$, -(OH)${}_{2}$, -CO${}_{2}$H) crystallizing in the MIL-53-type structure, it was recently exhibited that NH${}_{2}$-MIL-53(Al) did not present structural transition, which explains the little attention given to its flexibility [27,28], although Ahmfelt and coworkers have found similar breathing behavior to that unfunctionalized MIL-53 [29]. Herein, we computationally investigate then, for the first time, the effect of the temperature and the adsorption of a polar guest (methanol) on the structure and the flexibility of the NH${}_{2}$-MIL-53(Al). Let us mention that, although the comparison of the effect of the adsorption of polar and no polar molecules on the flexibility of NH${}_{2}$-MIL-53(Al) can be very interesting, it is not the scope in this work, and that is under investigation.

## 2. Materials and Methods

**Figure 1:**
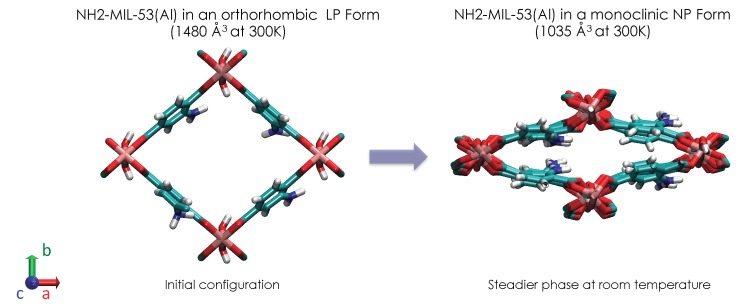
Illustration of the initial Narrow Pore (NP) and Large Pore (LP) forms of the NH${}_{2}$-MIL-53(Al) metal organic framework.

The simulation box was built from 32 unit cells of NH${}_{2}$-MIL-53(Al) along the *x*, *y* and *z* directions. The initial configuration was built by grafting the NH${}_{2}$ group in the ortho position of the benzenic cycles of the LP form of the MIL-53(Al) material. Atomic positions of the LP form correspond to the experimental coordinates previously obtained from X-ray powder diffraction [24]. An illustration of the LP-NH${}_{2}$-MIL-53(Al) is provided in Figure 1.

**Figure 2:**
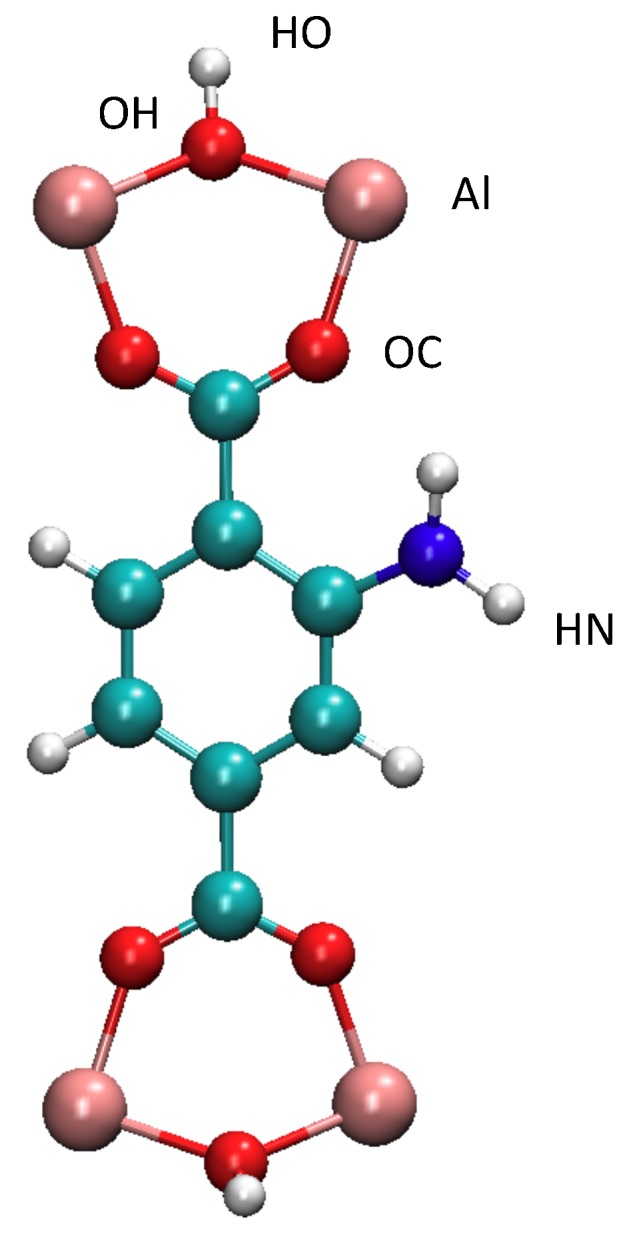
Cluster of atoms used in the charge calculation [34]. Labels of atoms (OH, HO, Al, OC, HN) implicated in the radial distribution functions’ calculation are also represented.

Experimentally speaking, it has been well known that the NH${}_{2}$-MIL-53(Al) is thermodynamically stable in the NP form [27,30]. We took the route to use the LP form as the initial configuration in order to ensure that our force field was capable of predicting the steadier phase. The initial box lengths correspond to the 32 unit cells of the LP-MIL-53(Al), *L*${}_{x}$ = 33.2 Å, *L*${}_{y}$ = 52.8 Å and *L*${}_{z}$ = 26.8 Å. All molecular dynamics simulations were performed by using the DLPOLY software (Version 4.0) [31]. The velocity-Verlet algorithm in the NσT statistical ensemble was used. N is the number of molecules, T the temperature and σ the isotropic constraint (1 bar). The Nose–Hoover thermostat and barostat was used with a relaxation time of 0.5 ps. Acquisition phases were performed during 5 ns by using a time step of 1 fs, whereas the equilibration phases lasted 2.5 ns. The Ewald summation was used for calculating the electrostatic interactions. Long- and short-range interactions were truncated at 12 Å. The NH${}_{2}$-MIL-53(Al) framework was considered as flexible and was modeled by refining the force field of the MIL-53(Cr) [19]. The force fields of the NH${}_{2}$ group and Al atom were considered by combining the DREIDING [32] and AMBER [33] models. Partial charges were taken in [34], where calculations were carried out on the cluster represented in Figure 2. Methanol molecules were modeled by means of the OPLS (Optimized Potentials for Liquid Simulations) force field [35] because it has been shown that the structure and physical properties of liquid and gas were well reproduced [35]. The van der Waals interactions between the NH${}_{2}$-MIL-53(Al) material and methanol molecules were calculated by using the mixing rules of Lorentz and Berthelot. Let us mention that the methanol molecules were randomly inserted. All parameters are provided in ForceFIELD.txt (the force field), CONFIG.txt (coordinates of the initial configuration) and CONTROL.txt (thermodynamics parameters and integration algorithms). These files are the input files of the DLPOLY [31] software.

## 3. Results and Discussion

**Figure 3:**
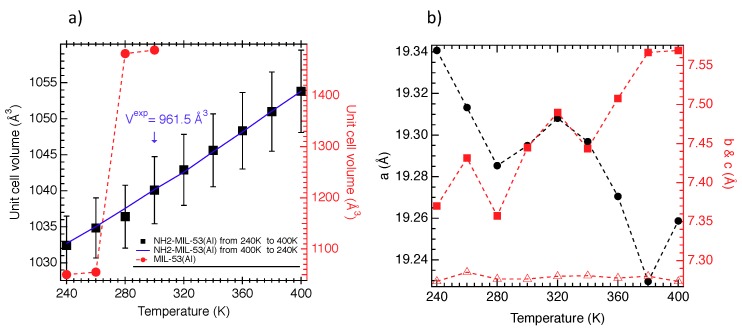
(**a**) Unit cell volume as a function of the temperature for the NH${}_{2}$-MIL-53(Al) (left axis) and for the MIL-53(Al) (right axis) materials; (**b**) Unit cell parameters; a (left axis), b (right axis) and c (right axis) as a function of the temperature for the NH${}_{2}$-MIL-53(Al) material. On average, unit cell parameters were calculated within a relative error of 0.2 Å.

We report in Figure 3a,b the unit cell volume (*V*${}_{u.c.}$) and the unit cell parameters (*a*, *b*, *c*) of the NH${}_{2}$-MIL-53(Al) as a function of the temperature. As shown in Figure 3a, *V*${}_{u.c.}$ slightly increases to 30 Å${}^{3}$ from 240 to 400 K. In this range of volume, the NH${}_{2}$-MIL-53(Al) is in the NP form. This result is in good agreement with the experiment where the LP form was not detected whatever the temperature [27]. Interestingly, a deviation of 8% of *V*${}_{u.c.}$ between simulation and experiment was found that was a source of force field validation. Additionally, the fact of finding a stable NP form that is in line with the experiment allowed us to be confident in the so-developed force field. Although the NP phase was systematically recovered, a slight increase in *V*${}_{u.c.}$ was observed that could suggest a possible reopening. As shown in Figure 3a, this evolution was fully reversible, and no hysteresis was observed. Let us note that at 500 K, an NP form of 1065 Å${}^{3}$ was found that bore out the continuous increase in *V*${}_{u.c.}$ and the possibility of a reopening. Actually, the thermal energy ($k_{B}T$ where $k_{B}$ is the Boltzmann constant) is not sufficient to activate the reopening. Probably the internal energy of the NH${}_{2}$-MIL-53(Al) has to be too high to be surpassed by the thermal one, which suggests strong intramolecular interactions. As shown in Figure 3a, a strong difference is observed with the MIL-53(Al) material because a structural LP↔NP transition is observed at 270 K. This difference between both materials can only be explained by the presence of the NH${}_{2}$ groups and is probably the result of the interactions between NH${}_{2}$ and other atoms such as oxygen and hydrogen atoms of the terephthalate moiety. Furthermore, Figure 3b shows that the increase in *V*${}_{u.c.}$ is due to the increase in the b value, whereas a decreases, and c is almost kept constant. This evolution of the unit cell parameters could suggest a possible reopening because it has been shown that the transition between the NP and LP phase is connected to the decrease of a, the increase of b and the quasi-conservation of c (see Table 1 of [19]).

**Figure 4:**
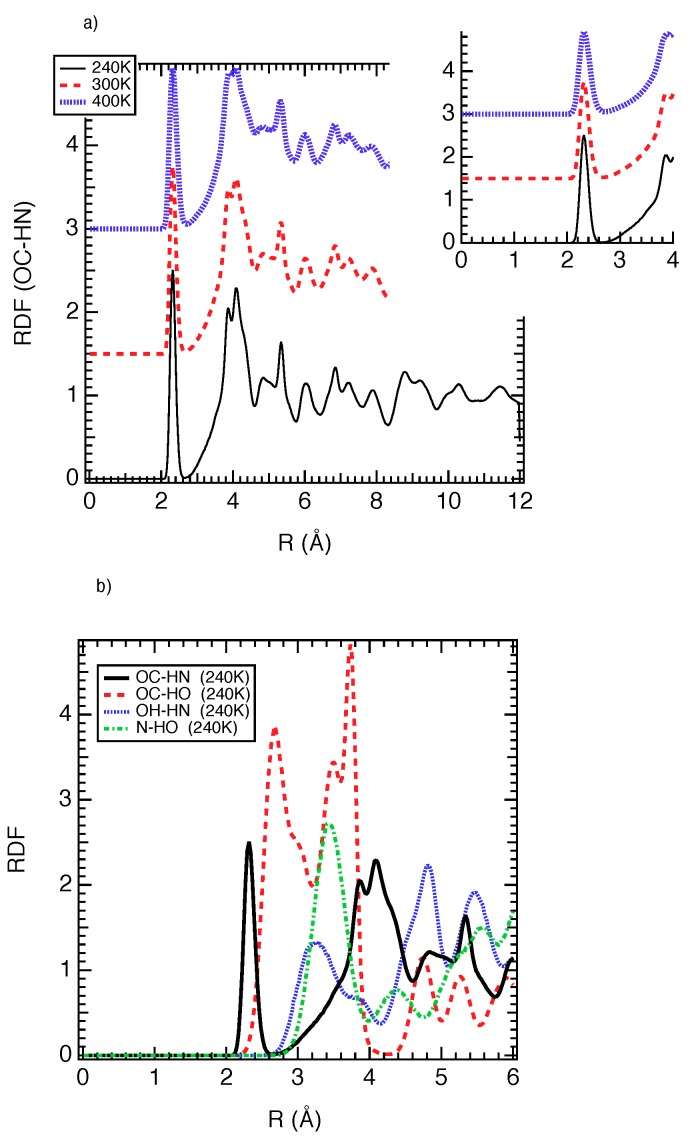
(**a**) Radial distribution functions between OC and HN (see Figure 2 for labels definition) at three temperatures. In the inset on top right an enlargement for the short distances was provided; (**b**) Radial distribution functions between OC and HN, OC and HO, OH and HN and N and HO at 240 K.

We manage in Figure 4a the radial distribution functions (RDF) between the oxygen atoms of carboxylate groups (labeled as OC in Figure 2) and the hydrogen atoms of NH${}_{2}$ groups (named HN in Figure 2). For a central particle, RDF describes how the local density evolves as a function of distance. From statistical physics, RDF corresponds to the probability of finding an atom at a distance of r from a central atom. As shown in Figure 4a, the first peak is located at 2.4 Å from 240 to 400 K, which involves strong intramolecular interactions of the same order of magnitude of a hydrogen bond that is located at 2.5 Å. This high affinity between OC and HN is probably at the origin of the stability of the NP form from 240 to 400 K. The RDF between OC and HO (see Figure 2 for the definition of labels) is also reported in Figure 4b. As exhibited in Figure 4b, the first peak is located around 2.6 Å, which suggests an interaction weaker than that of OC-HN, but strong enough to contribute to the stability of the NP phase. This OC-HO interaction suggests that the alumina octahedron (Al(OC)${}_{4}$(OH)${}_{2}$) could be slightly deformed. We calculated then the average angles of the octahedra, OC-Al-OC, OC-Al-OH and OH-Al-OH. We found $\langle ∠\mathrm{OC}-\mathrm{Al}-\mathrm{OC}\rangle$= 175.8${}^{\circ }$ and 90.2${}^{\circ }$, $\langle ∠\mathrm{OC}-\mathrm{Al}-\mathrm{OH}\rangle$= 88.8${}^{\circ }$ and $\langle ∠\mathrm{OH}-\mathrm{Al}-\mathrm{OH}\rangle$ = 178.2${}^{\circ }$. These values are close to the ideal octahedra, 90${}^{\circ }$ and 180${}^{\circ }$, which highlights an undeformed octahedral geometry. Additionally, let us note that the distance of 3.0 Å between OC and HO is also recovered in the LP of the MIL-53(Al), resulting in that this interaction cannot be at the origin of the stability of the NP form of NH${}_{2}$-MIL-53(Al). Therefore, the stable NP form is the result of the strong intramolecular hydrogen bonds between HN and OC. Additionally, as shown in Figure 4b, the interactions between OH and HN and N and HO seem to not take part in the stabilization of the NP form.

**Figure 5:**
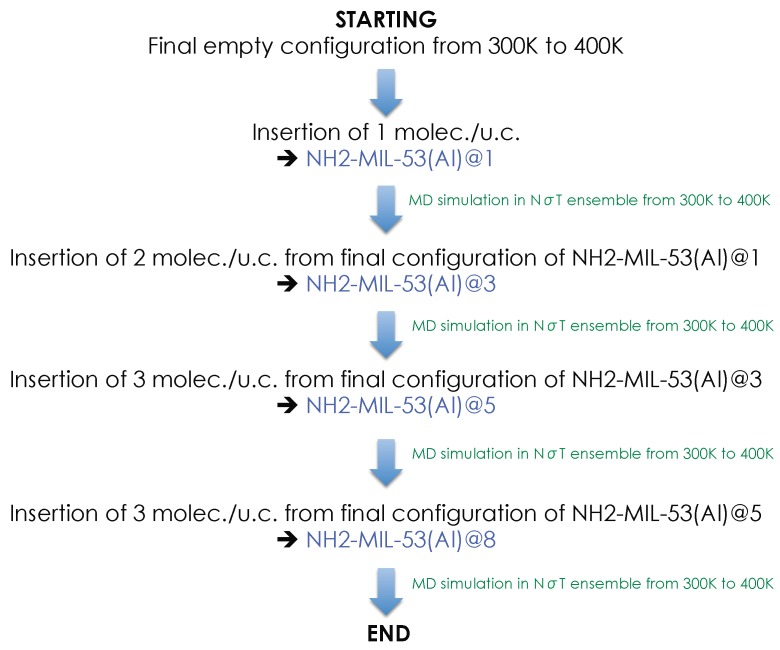
Scheme illustrating our protocol to evaluate the reopening of NH${}_{2}$-MIL-53(Al). molec./u.c., molecules by unit cell.

These results obviously bear out that the empty NH${}_{2}$-MIL-53(Al) is structurally and energetically stable under its NP form from 240 to 400 K. To assess the possibility of a reopening of the evolution of *V*${}_{u.c.}$ upon adsorption of a polar compound, methanol (MeOH) was investigated. MeOH molecules were then progressively inserted in the empty configuration NP-NH${}_{2}$-MIL-53(Al) at 300 K. We began by carrying out molecular dynamics (MD) simulation of one molecule by unit cell (1 molec./u.c.), NH${}_{2}$-MIL-53(Al)@1 from 300 to 400 K. From the final configuration of NH${}_{2}$-MIL-53(Al)@1, additional MeOH molecules were inserted to reach 3 molec./u.c., NH${}_{2}$-MIL-53(Al)@3. As shown in Figure 5, this process was repeated to finally fill the material at 8 moles./u.c.

**Figure 6:**
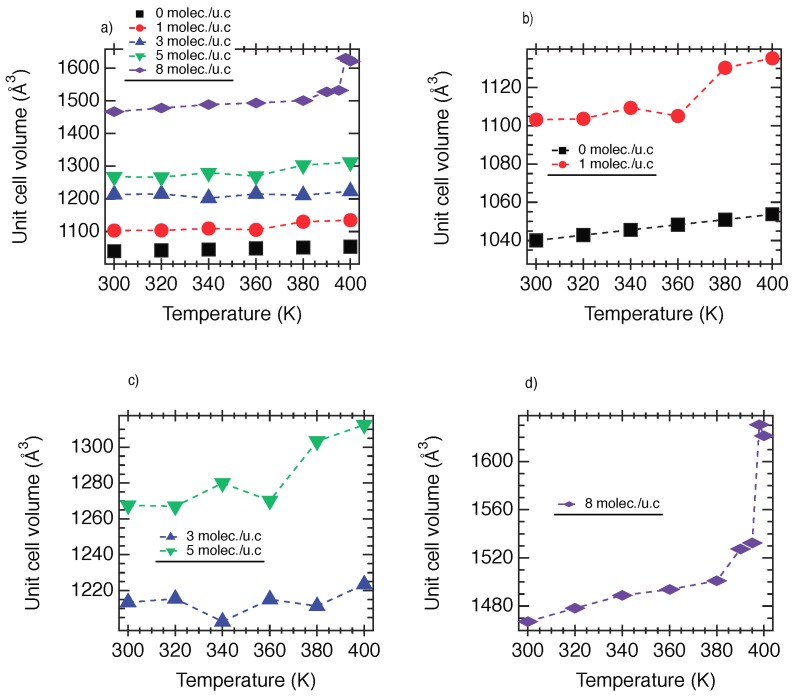
(**a**) Unit cell volume as a function of the temperature for 0, 1, 3, 5 and 8 molec./u.c.; (**b**) Unit cell volume as a function of the temperature for 0 and 1 molec./u.c.; (**c**) for 3 and 5 molec./u.c. and (**d**) for 8 molec./u.c.

**Figure 7:**
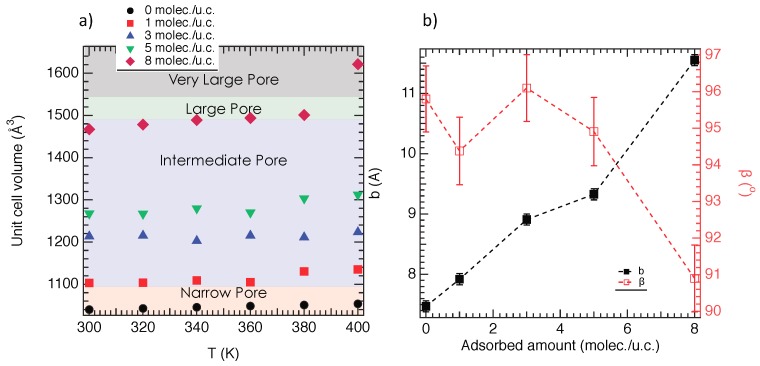
(**a**) Unit cell volume of the NH${}_{2}$-MIL53(Al) as a function of the temperatures and adsorbed amount; (**b**) Unit cell parameters (b and β) as a function of the adsorbed amount at 300 K.

We report in Figure 6 the unit cell volume as a function of the temperature for all cases. As shown in Figure 6a, for NH${}_{2}$-MIL-53(Al)@1, an increase of 6% of *V*${}_{u.c.}$ with respect to the empty material is observed. From an overall point of view, Figure 6a exhibits a progressive increase of *V*${}_{u.c.}$ from 1 to 8 moles./u.c.. As reported in Figure 6, the unit cell volume evolves from 1000 Å${}^{3}$ (in the empty phase) to 1600 Å${}^{3}$ via intermediate volumes, 1100 Å${}^{3}$, 1200 Å${}^{3}$, 1300 Å${}^{3}$, 1450 Å${}^{3}$. These results highlight that NH${}_{2}$-MIL-53(Al) can be opened upon adsorption. Interestingly, Figure 6b,c shows that the reopening does not continue because a transition is observed between 360 K and 370 K for both NH${}_{2}$-MIL-53(Al)@1 and NH${}_{2}$-MIL-53(Al)@5. The additional reopening is then facilitated by a thermal activation that increases the local intramolecular disorder and triggers an additional increase in unit cell volume. Unexpectedly, Figure 6d shows a large increase in *V*${}_{u.c.}$ for NH${}_{2}$-MIL-53(Al)@8. Indeed, a very large pore of a unit cell volume of 1600 Å${}^{3}$ is observed. This over-opening is the result of the combination of the strong steric effect of a large amount of methanol molecules and thermal energy involving an increase in the intramolecular motion. Thermodynamically speaking, both of these contributions induce a destabilization of the structure and trigger a transition toward a steadier phase. The domain of existence of these phases (narrow, intermediate, large and very large pore) with respect to the temperature is highlighted in Figure 7a.

Actually, the swelling of NH${}_{2}$-MIL-53(Al) can be imputed to a steric effect due to the MeOH molecules, which increase the pore size. As shown in Figure 1, the pore size of NH${}_{2}$-MIL-53(Al) can be quantified by the the unit cell parameter along the y direction, i.e., b. We report then in Figure 7b the b value as a function of the adsorbed amount. As shown in Figure 7b, an increase in b is observed, which suggests an opening of pore as a function of the adsorbed amount. Interestingly, Figure 7b also depicts that these intermediate phases present an NP symmetry, i.e., monoclinic. However, from 8 moles./u.c., the orthorhombic symmetry, i.e., the LP symmetry, is recovered.

**Figure 8:**
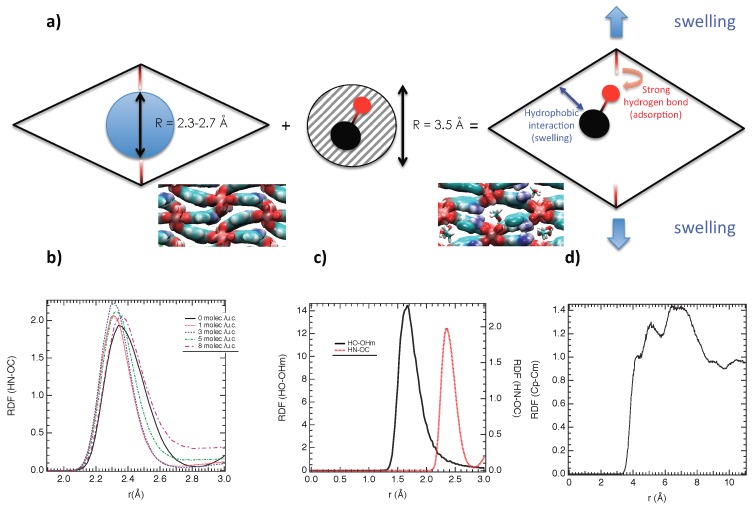
(**a**) Scheme illustrating the swelling from the adsorption of the MeOH molecule in the empty NH${}_{2}$-MIL53(Al); (**b**) Radial distribution functions between HN and OC for 0, 1, 3, 5 and 8 molec./u.c. at 300 K; (**c**) Radial distribution functions between the oxygen atom of the hydroxyl group of MeOH (Om) and HO and between HN and OC for 8 molec./u.c. at 300 K; (**d**) RDF between the carbon atoms of the benzenic groups of the MOF (Cp) and the carbon atoms of MeOH (Cm) at 300 K.

To capture the microscopic insight into the reopening, the RDF between HN and OC are reported in Figure 8b for 0, 1, 3, 5 and 8 molec./u.c. at 300K. As shown in Figure 8b, the location of the first peak that represents the strength of the interaction (short distances involve strong interactions) is slightly modified in the presence of MeOH and during the reopening. Furthermore, the amplitude of the first peak is also un-modified, which suggests that the HN-OC interaction takes place whatever the loading. The reopening is probably due to a steric effect such that the adsorbed MeOH exerted an internal pressure that surpasses the interactions at the origin of the closed structure [16] (which is illustrated in Figure 8a). The kinetic radius of MeOH is around 3.5 Å, whereas the pore radius of the empty material was evaluated at 2.5 to 2.7 Å (this value was obtained from the calculation of the Connolly volume of one pore). To obtain an adjustment between the pore radius of NH${}_{2}$-MIL53(Al) and the kinetic diameter of MeOH, the swelling is triggered.

Additionally, as shown in Figure 8c, the adsorption of MeOH into the NH${}_{2}$-MIL53(Al) is energetically favorable given the strong hydrogen bond interaction between the hydroxyl group of MeOH and the OH group of NH${}_{2}$-MIL-53(Al), which is in favor of its adsorption. Indeed, Figure 8c shows an interaction located at 2.4 Å, i.e., a shorter distance than HN-OC (2.8 Å). At the same time, the methyl group of MeOH (labeled Cm) is in a hydrophobic interaction with the carbon atoms of the benzenic cycles (named Cp). Indeed, as shown in Figure 8d, a distance of 4.5 Å was found as the location of the first peak, which is in line with the typical hydrophobic interaction. Actually, these hydrophobic interactions are in favor of the swelling of NH${}_{2}$-MIL-53(Al).

## 4. Conclusions

In this work, the thermal structural transition of the NH${}_{2}$-MIL-53(Al) metal organic framework was investigated by means of molecular dynamics simulations. The force field of the NH${}_{2}$-MIL-53(Al) was developed by refining the force field of the MIL-53(Cr). For the empty material, the NP form of the NH${}_{2}$-MIL-53(Al) was found to be steadier, in good agreement with the experiment. This stability and the absence of reopening are the result of the strong affinity between the oxygen atoms of the carboxylate groups and the hydrogen atoms of the amino groups. We showed that the adsorption of methanol triggered a progressive reopening of the structure by generating intermediate phases. We highlighted that this reopening is the result of a combination between hydrophobic interactions and strong hydrogen bonds. In future work, the differences between the influence of methanol and other molecules, on the one hand, water and, on the other hand, molecules that are not capable of hydrogen bond formation, for example dichloromethane or methane, will be investigated.
